# Clinical Judgement of Comfort in Preterm Infants by Neonatal Intensive Care Unit Nurses Accurately Reflects Neonatal Stress Exposure

**DOI:** 10.1055/a-2788-1968

**Published:** 2026-02-13

**Authors:** Nienke H. van Dokkum, Erik Koning, Alexia Lani, Helene A. Bouma, Arend F. Bos

**Affiliations:** Division of Neonatology, Department of Pediatrics, University Medical Center Groningen, University of Groningen, Beatrix Children's Hospital, Groningen, The Netherlands

**Keywords:** prematurity, NICU nurses, comfort assessment, neonatal stress exposure

## Abstract

**Objective:**

We aimed to investigate whether there was a correlation between the clinical judgement of comfort by neonatal intensive care unit (NICU) nurses and the daily stress exposure in the first 28 days of life in extremely to very preterm infants born before 30 weeks' gestation.

**Study Design:**

We included 45 infants born <30 weeks' gestation and/or <1,000 g of birth weight in an observational cohort study. Nurses' clinical judgement of comfort was based on a 10-point numeric rating scale. We quantified exposure to neonatal stress using the Neonatal Infant Stressor Scale. We calculated Spearman's correlation coefficients to determine the strength of the association between the two for each day between birth and 28 days of life.

**Results:**

Average clinical judgement scores ranged from 4 to 10, with means mainly between 7 and 8. Days 1 to 3 were excluded because of missing data. For days 4 to 20, we found moderate to high correlation coefficients. After day 21, we did not identify significant correlations anymore, possibly due to less variance in data.

**Conclusion:**

Clinical judgment of comfort in preterm infants by NICU nurses adequately reflects neonatal stress exposure. Our findings call for more research into the best measure of neonatal stress that encompasses both exposure to stressors and experience of stress.

**Key Points:**

- Measuring neonatal stress is difficult, and exposure may not be the same as experience.
- NICU nurses' judgement of stress and comfort accurately reflects exposure to stress.
- Exposure to stress and experience of stress are therefore related.
- Future studies should focus on creating a gold standard for measuring neonatal stress.

## Introduction

Nearly 10% of infants worldwide are born preterm, accounting for a total of 14 million infants. 1 Over the last decades, survival rates of preterm infants have drastically increased with advances in neonatal care. 1 Still, preterm infants, and especially those born very to extremely preterm below 30 weeks' gestation, 2 remain at higher risk for short-term and long-term adverse outcomes. Short-term adverse outcomes entail neonatal morbidities such as necrotizing enterocolitis, bronchopulmonary dysplasia, and intraventricular hemorrhages. 2 3 4 5 6 Long-term adverse outcomes entail poorer neurodevelopment, socioemotional development, and regulatory issues up to and including adolescence. 2 3 4 5 6

Adverse outcomes in the short-term and long-term may partly be explained by damage to the still developing brain. At 20 weeks’ gestation, the human brain only weighs 10% of the term brain, and cortical volumes as well as myelinated white matter drastically increase during this period, illustrating the importance of the second half of pregnancy for brain growth and development. 7 After birth, synaptogenesis and further maturation of the brain continues until reaching its adult size by 2 years of age, 7 making the neonatal period a sensitive time as well. During this sensitive time, preterm infants are admitted to the life-saving neonatal intensive care unit (NICU).

During NICU stay, preterm infants are subjected to neonatal stress, which can be defined as “adverse circumstances that disturb, or are likely to disturb, the normal physiological or psychological functioning of an individual.” 8 Among these are skin-breaking procedures, that most studies have focused on as pain-related stress, but also other physical stressors including medical and nursing procedures, separation from parents, as well as exposure to noise and light. 9 10 Stress exposure in the neonatal period significantly affects long-term neurodevelopment, a phenomenon that may be mediated by changes in immune and autonomic nervous system functioning, the hypothalamic–pituitary–adrenal (HPA) axis, and gene expression. 9 10 Additionally, neurodevelopment is moderated by the prenatal environment and maternal interaction. 9 10

Neonatal stress may be difficult to measure, because a gold standard is not yet available. Exposure to stressors may be quantified using a scoring sheet such as the Neonatal Infant Stressor Scale (NISS), a validated and standardized instrument designed to include skin-breaking procedures and other stressors that occur during NICU stay, to encompass the broad concept of neonatal stress. 11 The tool correlates well with biological cortisol samples 12 and neurobehavior. 13 However, exposure may not accurately reflect experience of stressors by preterm infants. Other measures, such as physiological signs or assessment scales may only reflect a single moment during a day. NICU nurses caring for preterm neonates play a pivotal role in the clinical team, serving as the constant presence at the bedside. Through their close observation and expert clinical judgement, they are uniquely positioned to assess signs of stress and comfort, acting as essential partners in guiding medical decision-making. As measuring neonatal stress is complicated, a comfort assessment by NICU nurses may serve as a first indicator of average or longer-term stress experience. If their clinical judgement reliably mirrors actual stress exposure, this could offer a practical, low-burden approach to monitoring stress in the NICU setting. However, it remains unclear whether these subjective assessments correspond to objective measures of stress exposure. The aim of this study was therefore to examine whether the clinical judgement of comfort by NICU nurses is associated with daily NISS scores during the first 28 days of life, being the early sensitive neonatal period, in extremely to very preterm infants born before 30 weeks' gestation. By exploring this association, we seek to better understand the reliability of nurse-based assessments as indicators of cumulative stress exposure in this vulnerable population.

## Materials and Methods

### Setting and Population

This substudy was part of a larger observational cohort study called “Stress and Outcomes in NICU Graduates (STRONG)” that included infants born before 30 weeks' gestation and/or a birth weight below 1,000 g, between September 2019 and December 2020. All infants were admitted to our level IV NICU. All parents of eligible infants were asked to participate in the study in the first week after birth. Written informed consent was obtained from all participating parents. The study was approved by the Medical Ethical Review Board of the University Medical Center Groningen (approval no.: METc 2019/128) as well as registered online (identifier: ISRCTN62164166). The current findings regard a secondary analysis of previously collected data.

### Measures and Procedure

We assessed neonatal stress exposure using the NISS. 11 This NISS was calculated daily for the first 28 days as the sum of acute and chronic items. Acute items include, for example, skin-breaking procedures, imaging procedures, inserting a gavage feeding tube, or nursing procedures such as nappy changes. Chronic items include, for example, suffering from a systemic infection, recovering from surgery, receiving intravenous (IV) fluids, or receiving phototherapy. All items are weighted to be a little stressful (weighted factor 2 points) to extremely stressful (weighted factor 5 points). In our setting, all of these items are recorded as standard of care and afterward collected from electronic patient files. The research team scored the NISS for 24-hour intervals, except on the day of birth and day of discharge, where the NISS score was calculated until midnight or until moment of discharge, respectively. Acute and chronic items were combined into a single score. In addition, to determine whether we could identify which stressors reflected observed differences, we constructed four subcategories within the NISS. The categories were based on expert opinion within the research team. These categories were (1) nursing, that is nappy changes, cleaning of eyes and mouth, suctioning of the nose and mouth, flushing IV lines, weighing infants, inserting feeding tubes and removing infants from an incubator, (2) skin-breaking, that is inserting IV lines, blood sampling, inserting drains, heel pricks, lumbar punctures, and surgery, (3) monitoring and imaging, that is electrocardiograms, ultrasounds, scans, X-rays, and application of sensors for cerebral function monitoring, electroencephalograms, or near-infrared spectroscopy, and (4) medical morbidity-related, that is suffering from a local or systemic infection, receiving phototherapy, intubation, ventilation (both mechanical and noninvasive) and eye examinations. The NISS and its categories do not have a minimum or maximum score but is dependent on the clinical setting in which it is used. Our previous work includes trajectories of the NISS per gestational age category and was published separately. 14 In summary, median daily NISS scores ranged between 50 and 85, with a large variation. On average, the mean daily NISS scores was 66.4 (standard deviation: 8.7).

Clinical judgement by NICU nurses was collected for the first 28 days as well. Nurses were asked to indicate the general level of comfort of the infant they cared for during their shift on a nonvalidated numeric rating scale of 1 to 10, from least to most comfortable possible. Data were collected in a paper folder that was placed at the bedside shortly after birth, but after informed consent was obtained. NICU nurses work in 8-hour shifts at our center. Nurses' shifts coincided with the 24-hour intervals of the NISS scores. These shift scores were averaged to get a daily clinical judgement of the nurses' comfort score as an indicator of an average, longer-term, neonatal stress experience. These scores were not previously analyzed nor published.

### Statistical Analyses

First, we described participant characteristics using descriptive statistics. We also provide descriptive statistics for the clinical judgement scores. We checked data for normality using Q–Q plots and the Shapiro–Wilk test and used nonparametric tests because of non-normal distribution in both NISS and clinical judgement scores. Second, we performed correlation analyses for each individual day between the averaged clinical judgement score and the NISS using Spearman's correlation coefficients. To ensure swift interpretation, we inverted clinical judgement scores so that both NISS and clinical judgement scores have the same direction, i.e., higher scores indicate more stress exposure. Statistical tests were performed using SPSS software, version 28.0 (IBM statistics, Armonk, New York, United States). *p* -Values below 0.05 were considered statistically significant.

## Results

### Participant Characteristics

We present participant characteristics of our study sample in Table 1 , including characteristics for those that did not participate as comparison. Ninety-three infants were eligible for participation in this study, of whom 45 participated. Reasons for exclusion were being deceased before informed consent could be asked ( *n* = 6), language barriers ( *n* = 2), declined participation ( *n* = 15), and logistical reasons, including the coronavirus disease 2019 research stop at our department ( *n* = 25). Infants that did not participate in the study had a shorter admission to the NICU because they died more often, possibly due to a higher incidence of necrotizing enterocolitis. They were also more likely to be part of a multiple birth. No infants over 30 weeks' gestation were included in the study.

**Table 1:** Participant characteristics

	Participating infants ( *N* = 45)	Nonparticipating infants ( *N* = 48)
Gestational age (wk)	27 (26–28)	27 (26–29)
Birth weight (g)	1,000 (790–1,248)	1,030 (791–1,253)
Male sex	22 (48.9)	24 (50.0)
Multiple birth	9 (20.0)	21 (43.8) a
Apgar 1 min	5.5 (3.0–7.0)	5.0 (2.0–7.0)
Apgar 5 min	7.0 (6.0–8.0)	7.5 (6.0–8.0)
NICU admission for surviving infants (d)	35 (24–49)	18 (11–31) ^c^
Deceased during NICU admission	2 (0.4)	13 (27.1) ^b^
Delivery via cesarean section	23 (51.1)	20 (41.7)
Antenatal steroids	38 (84.4)	43 (89.6)
Complete course	23 (51.1)	31 (64.6)
IVH grade ≥ grade 3	6 (13.3)	5 (10.4)
Mechanical ventilation	30 (66.7)	32 (66.7)
Days ( *n* = 30)	7.0 (2.8–20.5)	4.0 (2.0–9.0)
NEC	4 (8.9)	12 (25.0) a
Sepsis	16 (35.6)	15 (31.3)
Circulatory insufficiency	5 (11.1)	9 (18.8)
PDA	20 (44.4)	15 (31.3)
Cumulative NISS score present		
At day 7	44 (97.8)	N/A
At day 14	43 (95.6)	N/A
At day 28	29 (64.4)	N/A

Abbreviations: IC, intensive care; IVH, intraventricular hemorrhage; N/A, not applicable; NEC, necrotizing enterocolitis; NICU, neonatal intensive care unit; NISS, Neonatal Infant Stressor Scale; PDA, patent ductus arteriosus.

Notes: Data are presented as median (25th–75th percentile) or *N* (%) where appropriate. Complete course of antenatal steroids was defined as birth >48 hours after the first dose. Presence of intestinal pathologies was based on clinical and radiographic examinations. Sepsis was defined as clinical signs of infection, combined with a positive blood culture and requiring antibiotic treatment. Circulatory insufficiency was defined as requiring fluid therapy and/or treatment with inotropic agent, such as dopamine or dobutamine. Reasons for missing scores were one child being born outside our hospital and transferred on day 3 after birth and one infant being discharged to a post-IC unit on day 12 after birth and 14 other infants were discharged to a post-IC unit before day 28 after birth. In the nonparticipating group, six of these infants were already deceased before consent could be asked, six of them deceased during the coronavirus disease 2019 research stop and for the one other infant there was a language barrier for consent.

a *p* < 0.05, ^b^ *p* < 0.01, ^c^ *p* < 0.001 for nonparticipating versus participating infants.

### Clinical Judgement of Comfort by Neonatal Intensive Care Unit Nurses

We decided to exclude days 1 to 3 from our analyses, because of a large amount of missing data, inherent to the study design, where informed consent was asked in the first few days after birth, and the paper folder for clinical judgement scores was placed at the bedside thereafter. For the other days, we missed scores for 1 to 17 infants. After 21 days, the scores were missing for relatively more infants, because they were transferred to a postintensive care or high care ward in a regional hospital, as is standard practice in the Netherlands. Average scores ranged from 4 to 10, with means mainly between 7 and 8. These descriptive statistics are displayed in Table 2 . Between days 4 and 21, we calculated correlation coefficients. In 13 out of these 18 days the clinical judgement scores and the NISS scores were statistically significantly correlated. Coefficients were between 0.3 and 0.6, thereby being moderate to strong associations. For another 2 out of these 18 days, correlation coefficients tended to be statistically significantly correlated ( *p* = 0.064 and 0.085), with correlation coefficients of 0.25. After day 21, we did not find statistically significant correlations, and coefficients were generally between 0.01 and 0.02. All correlation coefficients are presented in Table 3 . In the subcategories of the NISS, we found correlations as well with similar strengths.

**Table 2:** Descriptive statistics of clinical judgement scores by neonatal intensive care unit nurses for days 4 to 28

Day	*N* a	Mean	Standard deviation	Median	Range	Min	Max
4	39	7.65	0.55	7.66	2.50	6.50	9.00
5	41	7,84	0.57	8.00	3.00	6.00	9.00
6	40	7.89	0.68	8.00	3.67	5.67	9.33
7	40	7.77	0.66	8.00	2.33	6.67	9.00
8	43	7.83	0.54	8.00	3.00	6.00	9.00
9	44	7.69	0.63	8.00	2.67	6.33	9.00
10	43	7.84	0.78	8.00	3.83	5.50	9.33
11	44	7.84	0.60	8.00	2.67	6.33	9.00
12	42	7.58	0.95	7.66	5.33	4.00	9.33
13	41	7.78	0.67	8.00	4.00	5.50	9.50
14	43	7.67	0.79	8.00	3.33	6.00	9.33
15	40	7.75	0.68	7.66	3.33	5.67	9.00
16	41	7.81	0.88	8.00	4.33	5.00	9.33
17	41	7.71	0.64	7.66	2.50	6.50	9.00
18	41	7.93	0.74	8.00	3.00	6.00	9.00
19	39	7.81	0.53	8.00	2.33	6.67	9.00
20	36	7.76	0.65	8.00	3.33	6.00	9.33
21	35	7.59	0.81	7.66	4.00	5.00	9.00
22	36	7.79	0.68	8.00	3.00	6.00	9.00
23	34	7.78	0.65	7.83	2.50	6.50	9.00
24	33	7.84	0.58	8.00	2.33	6.67	9.00
25	30	7.88	0.57	8.00	2.00	7.00	9.00
26	28	7.95	0.53	8.00	2.00	7.00	9.00
27	28	7.95	0.49	8.00	2.00	7.00	9.00
28	29	7.94	0.56	8.00	3.00	7.00	10.00

Abbreviation: NICU, neonatal intensive care unit.

a Number of infants with available clinical judgement scores data.

**Table 3:** Spearman's correlation coefficients between clinical judgement score by neonatal intensive care unit nurses and neonatal infant stressor scale

Day	*N* ^e^	NISS total	NISS nursing procedures	NISS skin-breaking procedures	NISS monitoring-imaging procedures	NISS morbidity related
4	39	0.087	0.140	−0.141	0.103	−0.139
5	41	0.139	0.032	0.317 a	0.247	−0.047
6	40	0.429 ^b^	0.258	0.412 ^b^	0.332 a	0.234
7	40	0.468 ^b^	0.306 ^d^	0.134	0.177	0.393 a
8	43	0.285 ^d^	0.258 ^d^	0.107	0.035	0.515 ^c^
9	44	0.328 a	0.208	0.271 ^d^	0.295 ^d^	0.431 ^b^
10	43	0.436 ^b^	0.313 a	0.461 ^b^	0.388 a	0.403 ^b^
11	44	0.434 ^b^	0.333 a	0.223	0.258 ^d^	0.253 ^d^
12	42	0.517 ^c^	0.285 ^d^	0.353 a	0.239	0.377 a
13	41	0.453 ^b^	0.460 ^b^	0.277 ^d^	0.134	0.294 ^d^
14	43	0.520 ^c^	0.424 ^b^	0.544 ^c^	0.423 ^b^	0.328 a
15	41	0.119	0.010	0.358 a	0.360 a	0.131
16	41	0.611 ^c^	0.542 ^c^	0.493 ^b^	0.400 a	0.397 a
17	41	0.426 ^b^	0.362 ^b^	0.294 ^d^	−0.013	0.412 ^b^
18	41	0.395 a	0.268 ^d^	0.310 a	0.465 ^b^	0.407 ^b^
19	39	0.279 ^d^	0.225	0.306 ^d^	0.434 ^b^	0.155
20	36	0.437 ^b^	0.113	0.470 ^b^	0.248	0.683 ^c^
21	35	0.483 ^b^	0.300 ^d^	0.473 ^b^	0.335 a	0.436 ^b^
22	36	0.264	NA	NA	NA	NA
23	34	0.229	NA	NA	NA	NA
24	33	0.141	NA	NA	NA	NA
25	30	0.169	NA	NA	NA	NA
26	28	0.056	NA	NA	NA	NA
27	28	0.151	NA	NA	NA	NA
28	29	0.300	NA	NA	NA	NA

Abbreviations: IV, intravenous; NA, not applicable; NICU, neonatal intensive care unit; NISS, neonatal infant stressor scale.

Note: Categories: (1) nursing, that is nappy changes, cleaning of eyes and mouth, suctioning of the nose and mouth, flushing IV lines, weighing infants, inserting feeding tubes and removing infants from an incubator, (2) skin-breaking, that is inserting IV lines, blood sampling, inserting drains, heel pricks, lumbar punctures, and surgery, (3) monitoring and imaging, that is electrocardiograms, ultrasounds, scans, X-rays, and application of sensors for cerebral function monitoring, electroencephalograms, or near-infrared spectroscopy, and (4) medical morbidity-related, that is suffering from a local or systemic infection, receiving phototherapy, intubation, ventilation (both mechanical and noninvasive) and eye examinations.

a *p* < 0.05, ^b^ *p* < 0.01, ^c^ *p* < 0.001, ^d^ *p* < 0.10, ^e^ number of infants with available clinical judgement scores data.

## Discussion

This study aimed to investigate whether there was an association between clinical judgement of comfort by NICU nurses and the daily NISS scores in the first 28 days of life in extremely to very preterm infants. Our findings demonstrated that clinical judgement scores were moderately to strongly associated with NISS scores for each day during the first 3 weeks of life, but not in the fourth week of life. Additionally, all subcategories of the NISS score, i.e., nursing procedures, skin-breaking procedures, monitoring and imaging procedures, and medical morbidity-related procedures contributed more or less equally to this association.

We found that clinical judgement assessed by nurses accurately reflects neonatal stress exposures. In addition, all parts of the NISS are reflected in the clinical judgement scores. Thus, the categories of skin-breaking procedures, medical procedures, monitor and imaging and nursing procedures all correlate to overall comfort as judged by NICU nurses. This indicates that not only pain-related stress, but other stressful events as well, are taking into account by experienced NICU nurses when they rate comfort of their patients. While the clinical judgement scores of nurses were an average of three shifts, they coincided with the NISS intervals of 24-hour scores and may therefore reflect an average and longer-term indication of stress experience that can be related to the same period of stress exposure. To our knowledge, this is the first study examining such an association. It is a core task of a NICU nurse to observe a preterm or sick infant and assess their comfort. Usually, comfort assessment is done using clinical scales assessing pain or distress. 15 These scales typically include physiological indicators such as heart rate, blood pressure, respiratory rate and oxygen saturation, behavioral indicators such as crying, facial expressions and behavioral states, and contextual indicators such as gestational age or postmenstrual age. 15 Even though all of these may adequately reflect distress at any given point in the day, they do not reflect overall comfort. A simple numeric rating scale now seems indicative of overall comfort for preterm infants.

Interestingly, we did not identify correlations during the fourth week of NICU stay between clinical judgement scores and NISS scores. This may be explained by a combination of factors. First, the number of children decreased slightly because of transfers to regional high care treatment centers, as it is national policy in the Netherlands for all infants that have reached clinical stability with minimal respiratory support at a postmenstrual age of 30 weeks or higher and with a current weight of 1,000 g or more. Second, the children that still require intensive care treatment in the NICU may be stable, just not yet fulfilling the criteria for transfer. Third, neonatal stress exposure decreases over the first 28 days of life, with especially the fourth week having relatively little exposure left. 14 The minimal clinical judgement score increased at the same time, i.e., nurses scored less stress as well, thereby reducing the range of scores and its variance.

We compared our numeric rating scale to the NISS score. Counting stressful exposure during NICU stay has increasingly been used to indicate cumulative neonatal stress. 16 17 18 Others have used cumulative skin-breaking procedures as a proxy of neonatal stress. 9 The downside of both counting skin-breaking procedures, but also the NISS, is that it only measures exposure to stressful events, rather than how the infant experiences these stressful events. Other methods of assessing neonatal stress experiences could be measuring physiological or hormonal responses, for example, through heart rate variability as a measure of autonomic nervous system response, 19 20 or cortisol as a measure of the HPA axis' stress response. 12 21 However, these responses may only occur in a specific condition and may also be signs of deteriorating medical conditions. Other behavioral scales may reflect a single point in time as well. As such, a measure of average or longer-term stress experience is still highly needed but also needs to be evidence-based. By comparing the NISS to the nurses' clinical judgement scores, we now show that exposure and experience are tightly associated, and that clinical judgement assessed by NICU nurses may be a good indicator of overall comfort.

## Strengths and Limitations

The main strength of our study is the prospective collection of both NISS scores and nurses' clinical judgement scores. We also recognize our limitations. First, the number of participating infants was relatively small. In that respect, we were also unable to include days 1 to 3, because of the logistical setup of the study. With this in mind, findings may not be generalizable for all NICU settings. Additionally, the NISS is a consensus-based instrument, that is not psychometrically validated. However, studies do show that the NISS is correlated with more physiological markers of stress, 12 which makes it a valuable instrument. Second, we do not have information on the underlying reasons for a particular clinical judgement score. In hindsight, this could have provided more insight into what parameters nurses base their clinical judgement. We also do not have background characteristics on the nurses, which might hamper generalization of the results. As the study ran for a year, we believe the full unit participated in providing clinical judgement scores, which may increase generalizability. We do know that our sample is of limited diversity regarding ethnicity, which should be taken into account when interpreting these results. Finally, the clinical judgement scores of nurses were based on a simple numeric rating scale, which may not fully capture stress experience, but serves as a first step toward better assessment.

## Implications

Our findings call for more research into the best measure of neonatal stress that encompasses both exposure to stressors and experience of stress. NICU nurses should be part of such a discussion, as their clinical judgement is what physicians rely on. These studies should address the gaps, including a larger sample and a more detailed study of background characteristics of nurses. Additionally, for future research, we would like to include parental perspectives of neonatal stress as well. For example, in bereaved parents, research has shown that infant signs are well captured by parents. 22 23 They get to know their newborn fast and may therefore also be a valuable source of information on longer-term or average stress experiences.

## Conclusion

Clinical judgment of comfort in preterm infants by NICU nurses adequately reflects neonatal stress exposure. Defining, measuring, and quantifying all aspects of neonatal stress in preterm infants during NICU stay remains an important quest that may need different tools depending on specific situations.
